# Retraction: Li, M., et al. W-GPCR Routing Method for Vehicular Ad Hoc Networks. *Sensors* 2020, *20*, 3406

**DOI:** 10.3390/s21061998

**Published:** 2021-03-12

**Authors:** Min Li, Zhiru Gu, Yonghong Long, Xiaohua Shu, Qing Rong, Ziji Ma, Xun Shao

**Affiliations:** 1 College of Traffic Engineering, Hunan University of Technology, Zhuzhou 412007, China; minli0103@126.com (M.L.); lyhcai@126.com (Y.L.); sxhdata@126.com (X.S.); Rong08017@163.com (Q.R.); 2 College of Electrical and Information Engineering, Hunan University, Changsha 410082, China; zijima@hnu.edu.cn; 3 Division of Information and Communication Engineering, Kitami Institute of Technology, Hokkaido 090-8507, Japan; x-shao@ieee.org

The journal retracts the article [1], cited above, published in the journal, *Sensors*. The authors contacted the publisher regarding the robustness of the results. The selection of weight parameters was not sufficiently rigorous, resulting in doubts concerning the scientific validity of the theoretical models and simulation results.

Adhering to our procedures, an investigation was conducted by the Editor-in-Chief with the editorial office of *Sensors*. The extent of the error is significant and, therefore, in accordance with the journal’s procedural requirements, this article is retracted.

The authors agreed to this retraction and apologize for any inconvenience caused.
